# Supplementary material for: Identification of VvAGL Genes Reveals Their Network’s Involvement in the Modulation of Seed Abortion via Responding Multi-Hormone Signals in Grapevines
Source: Int J Mol Sci. 2024 Sep 12;25(18):9849. doi: 10.3390/ijms25189849 (PMC11432271; doi:10.3390/ijms25189849)
Supplement: Supplementary file 1 [file ijms-25-09849-s001.zip › Supplementary Table S1.pdf]

Supplement Table S1

Table S1. Classification and properties of *VvAGL* gene family members

	GeneID	Chromosome location	Residual base	Molecular mass/KD	Isoelectric point	Fat factor	Hydrophilicity
Ⅰ	VIT_200s0250g00085	Chr0	969	81966.14	5.04	24.66	0.854
	VIT_207s0129g00650	Chr7	189	15161.67	5.40	35.98	0.762
	VIT_200s1450g00005	Chr0	966	78260.61	5.09	24.02	0.650
	VIT_201s0010g01500	Chr1	1439	121180.53	5.02	33.77	0.820
	VIT_201s0010g01530	Chr1	639	52511.36	5.17	27.54	0.715
	VIT_203s0088g00510	Chr3	1545	129481.465	5.05	30.61	0.603
	VIT_203s0088g00550	Chr3	642	52877.00	5.16	25.39	0.721
	VIT_203s0088g00590	Chr3	645	52974.35	5.16	29.30	0.815
	VIT_203s0088g00600	Chr3	645	52878.05	5.16	28.84	0.780
	VIT_203s0088g00610	Chr3	660	54598.75	5.17	29.09	0.736
	VIT_207s0005g06590	Chr7	662	53627.17	5.16	21.00	0.584
	VIT_210s0003g03015	Chr10	558	45763.11	5.20	32.97	0.832
	VIT_210s0003g03020	Chr10	585	48717.48	5.18	27.35	0.753
	VIT_210s0003g03025	Chr10	669	54051.21	5.14	21.08	0.677
	VIT_210s0003g03970	Chr10	673	54635.43	5.15	20.80	0.601
Ⅱ	VIT_200s0211g00110	Chr0	9314	787216.99	4.62	32.49	0.772
	VIT_200s0211g00180	Chr0	21426	1790799.22	4.47	32.24	0.679
	VIT_200s0250g00085	Chr0	1101	90801.66	4.99	27.97	0.691
	VIT_200s0729g00010	Chr0	644	56184.69	5.15	20.65	0.582
	VIT_202s0025g04650	Chr2	2291	191626.98	4.95	31.43	0.695
	VIT_203s0167g00100	Chr3	1064	92030.50	4.98	24.06	0.619
	VIT_208s0007g08790	Chr8	2189	184681.96	4.93	33.49	0.645
	VIT_213s0158g00100	Chr13	8037	675629.65	4.70	30.12	0.635
	VIT_214s0068g01800	Chr14	1367	116524.16	4.97	28.68	0.652
	VIT_215s0024g02000	Chr15	716	62557.81	5.13	21.09	0.587
	VIT_215s0048g01240	Chr15	1281	103949.65	4.97	25.37	0.644
	VIT_215s0048g01270	Chr15	17774	1595992.30	4.53	25.06	-0.009
	VIT_216s0022g02400	Chr16	1842	158661.43	4.92	23.29	0.544
	VIT_218s0041g01880	Chr18	8093	691918.67	4.68	30.52	0.680
Ⅲ	VIT_202s0109g00382	Chr2	1226	99761.91	5.06	25.04	0.615
	VIT_202s0109g00384	Chr2	894	72948.85	5.01	26.51	0.685
	VIT_203s0097g00192	Chr3	630	52346.19	5.21	36.35	0.813
	VIT_205s0020g01043	Chr5	708	56743.59	5.13	28.95	0.843
	VIT_205s0020g01046	Chr5	698	55918.32	5.15	28.37	0.785
	VIT_205s0020g01055	Chr5	6742	559148.76	4.68	32.63	0.847
	VIT_208s0032g00974	Chr8	1599	128250.06	5.02	26.83	0.643
	VIT_214s0060g00300	Chr14	711	57146.77	5.15	30.52	0.820
	VIT_215s0021g00560	Chr15	7194	605263.03	4.77	31.53	0.523
	VIT_215s0021g02220	Chr15	985	81824.66	4.99	32.49	0.838

	VIT_215s0021g02250	Chr15	1033	85198.15	4.99	32.91	0.823
