# Supplementary material for: Identification of VvAGL Genes Reveals Their Network’s Involvement in the Modulation of Seed Abortion via Responding Multi-Hormone Signals in Grapevines
Source: Int J Mol Sci. 2024 Sep 12;25(18):9849. doi: 10.3390/ijms25189849 (PMC11432271; doi:10.3390/ijms25189849)
Supplement: Supplementary file 1 [file ijms-25-09849-s001.zip › Supplementary Table S2.pdf]

Supplement Table S2

Table S2. qPCR primer sequences

Name	Forward primer(5′-3′)	Reverse primer(5′-3′)
VvAGL11	GCTCGAGAACAGGCTTGAAC	GCCTCTCCACTTCTGCAATC
VvAGL80	AGTGAGGTCGAACGAGTGCT	CCACTCAAACACTGGGTCCT
VvAGL6-2	TTGTGATGCTGAGGTTGCTC	CAGCGGTCCAAGATCTTCTC
VvAGL3	CAGCTGAAACGAATCGAGAA	ATTCGTAAAGCCTGCCTCTG
VvAGL15	TCCGGCTTGAGCTTAAAAGA	CAACTCGAAGCTCCTCAACC
VvAGL62	GAAGCGAGAGGTCCTTGATG	GCAACCTCCTTCTGCAACTC
